# Unraveling the Diagnostic Challenge of Arachnoiditis Ossificans in Association with Syringomyelia: A Review of the Literature and Two Case Reports

**DOI:** 10.1055/a-2740-7947

**Published:** 2025-11-17

**Authors:** Fatemeh Khafaji, Jochen Tuettenberg, Clemens Sommer, Bernardo Reyes Medina, Frank Hertel

**Affiliations:** 1 Department of Neurosurgery, Medical Campus Upper Franconia, Friedrich-Alexander-University Erlangen-Nuremberg, Bayreuth, Germany; 2 Department of Neurosurgery, SHG Klinikum Idar-Oberstein, Idar-Oberstein, Germany; 3 Institute of Neuropathology, University Medical Center of the Johannes Gutenberg-University Mainz, Mainz, Germany; 4 Department of Neurosurgery, Medical Campus Upper Franconia, Friedrich-Alexander-University Erlangen-Nuremberg, Bayreuth, Germany; 5 Department of Neurosurgery, Centre Hospitalier de Luxembourg, Luxembourg City, Luxembourg

**Keywords:** arachnoiditis ossificans, CSF block, syringomyelia

## Abstract

**Introduction:**

Arachnoiditis ossificans (AO) associated with syringomyelia (SM) is a rare pathology. Its clinical and image-based diagnostic features are challenging to identify. Only a limited number of cases have been published thus far. We present two new cases and offer a review of the literature.

**Materials and Methods:**

We conducted a systematic literature search using *PubMed* , *Web of Science* , and *Google Scholar* with the following keywords: Arachnoiditis ossificans, leptomeningeal calcification, and spinal meningeal calcification, in combination with syrinx, syringomyelia, hydromyelia, cord cavitation, and cystic necrosis of the spinal cord.

**Results:**

AO-SM predominantly affected females (12 F, 7 M), with a mean age of 55.84 ± 14.7 years. The mean follow-up was 14.07 ± 9.01 months postoperatively. The main complaints included low back pain and progressive para-/tetraparesis, with or without urinary disturbances. Potentially causative events occurred 25.07 ± 13.75 years prior to diagnosis. Based on imaging findings, patients primarily experienced thoracic AO-SM. In seven studies, arachnoid cysts were reported in association with AO-SM. Surgical treatment mainly involved microsurgical AO resection, shunting, or draining of the SM, along with duraplasty and cystectomy or fenestration of the arachnoid cyst. A second surgical intervention was conducted on five patients. Approximately 57% of the patients showed improvement.

**Discussion and Conclusion:**

AO-SM remains one of the least understood causes of myelopathy. Clinical and imaging diagnostics continue to pose challenges. Preoperative evaluation using magnetic resonance imaging (MRI) and native CT may be regarded as the gold standard. CT myelography and, occasionally, Cine MRI should be considered to determine the best surgical option. Surgical treatment continues to be a dilemma.

## Introduction

Arachnoiditis ossificans (AO) is a rare, end-stage form of adhesive arachnoiditis (AA), may result from trauma, infections such as meningitis, subarachnoid hemorrhage, or other inflammatory processes. The true incidence is unknown and can be mistaken for asymptomatic ossification of the spinal meninges, frequently found in autopsies. AO may cause CSF blockage and myelopathy leading to syringomyelia (SM), with a generally poor postoperative prognosis. The specific mechanisms underlying its development remain unclear. 1 2 Based on the radiological features proposed by Domenicucci et al in 2004, a practical classification of AO was established. Semicircular ossification was classified as type I, circular calcification encompassing the thecal sac was classified as type II, and caudal roots and entire sac content were classified as type III. 2

The exact mechanism of SM in association with AO is not known yet. Considering the chronic, slow natural course of AO-SM with its wide range of symptoms, makes it challenging to establish an accurate diagnosis. The coincidence of SM with AO was reported in 17 peer-reviewed case reports. Some authors have estimated that SM occurs in 13% of cases associated with AO. 3 Until 1975, before the invention of magnetic resonance imaging (MRI), the diagnosis was based on myelography. 4 Currently, diagnosis is based on MRI and at least a native CT scan. Eleven studies provided reports based on CT and MRI. Six studies used CT myelograms as a first-line or second-line diagnostic tool.

The surgical management of symptomatic patients with AO-SM varies widely. Numerous procedures have been reported, ranging from minimally invasive techniques like AO resection to more extensive options such as duraplasty, myelotomy, and/or SM shunting. However, only about 50% of patients experience a noticeable improvement after these surgeries. The long-term outcomes remain uncertain, as many studies lack comprehensive follow-up data. Establishing a definitive surgical approach is challenging due to the chronic nature of the disease.

This literature review provides a comprehensive overview of the pathophysiology of AO, its association with SM, possible differential diagnoses, classification of SM in association with AO, and available treatment options. Additionally, two case reports are included in this paper.

### Case 1

A 72-year-old woman was referred to our department due to progressive left-dominant spastic paraparesis, gait ataxia, low back pain, and numbness in her left leg, which had been worsening for the past 2 years. By the time of her presentation, she was having significant difficulty walking, even with the assistance of a walking aid. Further examination also revealed urinary retention. Her medical history included a severe case of meningitis at the age of 17, which resulted in right oculomotor nerve palsy. Fortunately, this condition resolved without any lasting neurological effects.

**Fig. 1:**
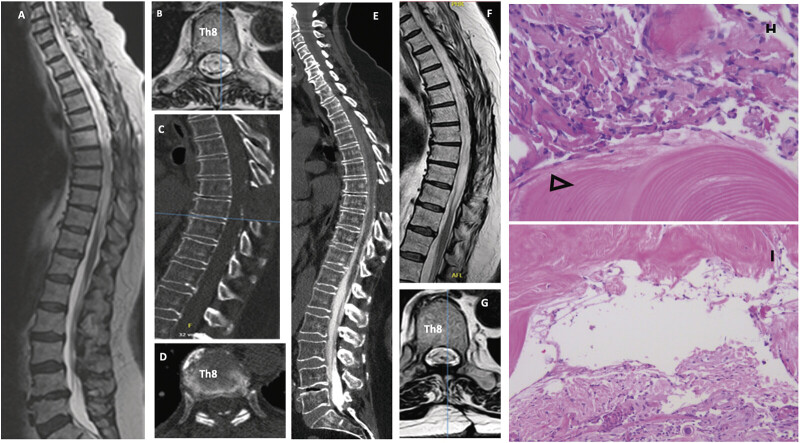
Imaging studies of patient 1. ( **A** ) Sagittal T2-weighted MR image showing long thoracic syringomyelia. ( **B** ) Axial T2-weighted MR image of Th 8 depicting hypointensity in the posterior portion of the dural sac and hyperintensity at the center of the spinal cord representing the syrinx. ( **C, D** ) Sagittal and axial native CT images after the first surgery depicting the Th 7–8 decompression region and curvilinear calcification posteriorly to the thecal sac. ( **E** ) The myelogram showed CSF blockage at Th 8. ( **F, G** ) After 1 year, the syringomyelia remained unchanged. ( **H** [×400], **I** [×200]) HE staining of the resection specimen from Th 7–8 demonstrates meningothelial cells within the resected leptomeninges with ossification along the margin of the specimen as well as scattered foci of calcification (black arrowheads), consistent with AO. AO, arachnoiditis ossificans.

On clinical examination, she had proximal lower extremity weakness on the left side. Hyperreflexia in both legs (left > right). No sensory impairment was caused by pinprick, joint position, vibration, pain, or temperature sensation. MRI revealed SM from Th 3 to Th 10. On sagittal projection, multinodular non-enhancing hypointense lesions were observed in T2-weighted images posterior to the thoracic spinal cord. A lumbar puncture revealed slightly elevated protein. Finally, surgical exploration, including biopsy, was performed. The patient underwent a laminectomy at Th 7–8. After opening the dura, the spinal cord was covered with a light grayߞyellowish hard shell, which appeared to adhere to the spinal cord's dorsal surface. A small piece was detached carefully via the microsurgical technique. In the end, the dorsal side of the spinal cord was visible. The histological findings revealed lamellar bone with dense fibrous tissue. No inflammation was present. Postoperatively, the patient developed worsening paraparesis and numbness below Th 10, so she could be mobilized by a wheelchair. A urinary catheter was placed due to severe urinary retention. A CT myelogram and a Cine MRT confirmed a caudal CSF block at the operation site. Native CT revealed extensive ossification predominantly posterior to the spinal cord, which extended rostrally and caudally to the operation site. Because of her progressive sensory-motor deficit, decompressive surgery was performed. She underwent a laminectomy from Th 5 to Th 10, including arachnoidolysis and duraplasty. At the end of the surgery, the CSF pathway was reestablished. During the postsurgical course, the patient was sent to a rehabilitation facility. Her neurological status at the time of discharge was unchanged. The first postoperative MRI after 6 weeks showed no change in the size of the syrinx. Clinically, however, the patient could walk for a short distance with an aid. The numbness also decreased. After 1 year, the syrinx remained unchanged on MRI. She could get up and walk on the walker slightly better than last year. The voiding dysfunction was intermittent, and there was hypoesthesia at the Th 10 level. The image and clinical presentation will remain unchanged at the last visit in 2023 ( Fig. 1 ).

### Case 2

A 72-year-old male was admitted in 2018 due to severe lower back pain, progressive paraparesis and sphincter disturbances, and hypoesthesia below the dermatome Th 8 for 3 months. In 2002, he suffered from severe spondylodiscitis at L 3/4 with an epidural abscess, arachnoiditis of the cauda equina, and myelitis of the conus medullaris. The infection occurred after epidural anesthesia. At this time, the patient was admitted because of urinary retention, distal paraparesis, and fulminant sepsis. After a diagnostic biopsy, the infection with *Staphylococcus aureus* was treated according to the antibiogram. Ten years later, the patient received conventional spinal cord test stimulation for chronic back and leg pain, predominantly on the right side. However, the test stimulation failed to have a positive effect, so the electrodes were removed. The neurological examination in 2019 revealed spastic paraparesis (strength of 3/5) with bilateral upward Babinski. MRI revealed Th 4 to Th 7 septated SM, non-enhancing hyperintensity dorsal to the spinal cord from Th 4, and a hyperintense lesion dorsal and ventral to the spinal cord at the Th 6/7 level, with mass effects and myelopathy signals at the Th 11/12 level in T2-weighted images. Exploratory surgery was performed at the Th 6/7 level. The patient underwent a laminectomy on Th 6/7. During the microsurgical operation, a grayish–yellowish layer covered the spinal cord. No CSF flow was observed. Intraoperative echography revealed SM beneath the shell. Some calcified sheets were also observed. The shell was removed carefully. At the end of the surgery, CSF flow was observed. Dense fibrous collagenous tissue with osseous fragments and a single psammoma body was observed histologically, and no inflammatory cells were found.

Postoperative MR revealed the collapse of the syrinx at the Th 6/7 level. Three months later, MRI demonstrated the re-expansion of syrinx Th 4 to 7 with intramedullary edema Th 2 to Th 8 and a hypointense lesion dorsal to the spinal cord up to Th 10. Clinically speaking, the patient complained of back pain, which radiated predominantly on the right side. Clinical examination revealed paresis of plantar flexion and extension on the right foot. The MRI 4 months later revealed that the multicavity syrinx had grown rostrally to Th 3. There was no change in myelopathy at the Th 11/12 level. Two months later, the patient was admitted because of massive pain and deteriorated gait ataxia, as well as bladder/bowel trouble, in addition to ascending numbness. MRI revealed cranial expansion of the syrinx with intramedullary edema to Th 2, but the other abnormalities remained unchanged. As a result, the patient underwent a second surgical intervention. The operation was performed from the old operation side with cranial expansion. Calcification was detected and removed from the dorsal spinal cord. The syrinx collapsed intraoperatively. Duraplasty was done. The histological findings revealed mineralization and osseous fragments with scar formation. The postsurgical MR image revealed a collapsed Th 6 to Th 8 syrinx. Clinically speaking, the patient reported regression of his pain and was discharged home.

**Fig. 2:**
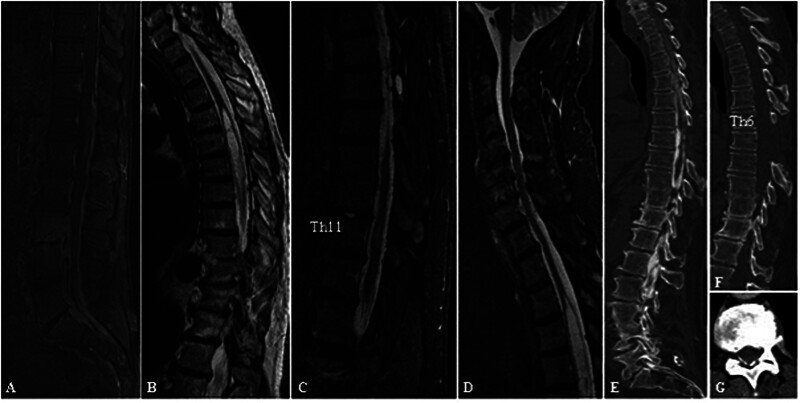
Image studies of patient 2. ( **A** ) Gadolinium contrast-enhanced MR image from 2002 showing spinal empyema. ( **B** ) Preoperative T2-weighted MR image showing the SM up to Th 4. ( **C, D** ) The MRI study 2019 revealed a re-expansion of the SM, regredience of the pseudomeningocele, and an unchanged myelopathy signal at Th 11. ( **E** ) Myelography performed in 2021 excluded CSF blockage. ( **G, F** ) A retrospective study in which the image depicted AO type II and I. AO, arachnoiditis ossificans; MRI, magnetic resonance imaging; SM, syringomyelia.

MRI at 3 months revealed a postsurgical pseudomeningocele at the Th 6–8 level. The SM remained stable until Th 3; unchanged myelopathy was observed at Th 11/12. The spastic paraparesis worsened. The patient received a cystocatheter for his incontinence. He was only able to go through some steps with walking aids. MRI performed in 2021 revealed regression of the pseudomeningocele and stability of other abnormalities. A retrospective review of the images showed a type II AO at the Th 11/12 level in a CT scan in 2018, corresponding to the myelopathy lesion at the Th 11/12 level. Multiple separated dorsal calcifications, representing type I AO, up to Th 4 dorsally, occasionally ventral to the spinal cord, and type III in the cauda equina. No CSF block was detected on CT myelography in 2021 ( Fig. 2 ).

## Materials and Methods

**Fig. 3:**
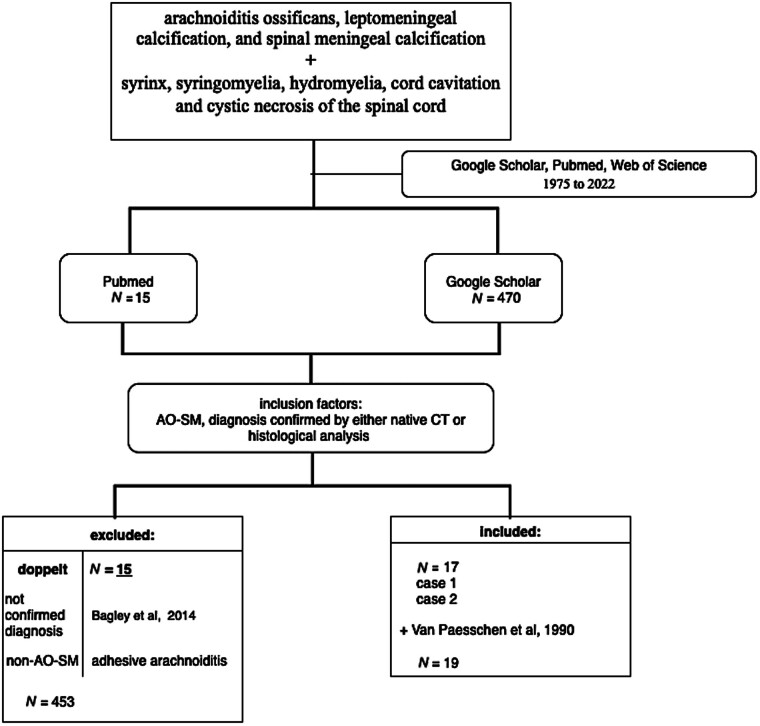
A schematic representation of the research methodology, including the articles that were excluded. AO, arachnoiditis ossificans; SM, syringomyelia.

Computerized search in English publications was performed through PubMed, Web of Science, and Google Scholar with the following keywords: Arachnoiditis ossificans, leptomeningeal calcification, and spinal meningeal calcification in combination with the following words: Syrinx, SM, hydromyelia, cord cavitation, and cystic necrosis of the spinal cord. From 470 articles identified in Google Scholar and 15 studies in PubMed, we included 17 patients with a coincidental finding of AO-SM (1975–2022). An AO-SM diagnosis was established utilizing MRI or myelography and confirmed by either native CT or histological analysis of the operative specimen, which revealed lamellar bone formation without an active inflammatory process. Patients with AA were excluded. A report by Van Paesschen et al, which demonstrated the transition of the chronic inflammatory process to ossification, was included because it met other criteria. 5 The report of Bagley et al was excluded because of the long-term existence of SM before AO diagnosis. 6 We included two patients who were treated in our two neurosurgical departments. Informed consent was obtained from both patients to publish the imaging studies and medical history ( Fig. 3 ).

## Results

Nineteen patients (7 male and 12 female, aged 30–81) were identified. The mean age ± SD was 55.84 ± 14.7 years (52.42 ± 17.85 and 57.83 ± 13.68 for males and females, respectively, *p* > 0.05). The mean follow-up time was 14.07 ± 9.01 months (range, 3–36 months). Most patients presented with low back pain, gait ataxia due to para-/tetraparesis, and paresthesia with or without urinary disturbance. The preceding possible causative events mentioned in the literature include meningitis, 2 7 tuberculous leptomeningitis, 5 spondylodiscitis, dorsal stabilization, 8 other spinal procedures, 9 10 subarachnoid hemorrhage, 11 12 13 sexually transmitted diseases, 4 trauma, 10 14 intradural phenol therapy, 10 posterior fossa operation, 3 spinal hemorrhage, 15 oil-based contrast myelogram, 15 and unknown. 12 16 17 18 The preceding events were identified only in 15 patients; these events occurred 25.07 ± 13.75 years before presentation (20.66 ± 9.02 and 28.37 ± 16.25 for males and females, respectively, *p* > 0.05, ranging from 8 to 55 years). Two studies reported the thoracolumbar AO. 7 15 Three studies did not include CT images of patients with unclear AO type, 3 4 9 and most patients experienced a thoracic type I or II AO according to the Domenicucci et al's classification. 2 SM was documented in seven patients with rostral expansion to AO localization, ranging from 1 to 8 segments, with a mean of 4 segments; five patients with caudal expansion, ranging from 1 to 6 segments, with a mean of 3.8 segments; two patients with rostrocaudal expansion; and four patients with a segment just as large as the AO. In seven patients, arachnoid cysts were also detected. 5 7 8 12 13 15 17 All patients underwent decompressive laminectomy; 13 patients (68%) had AO removal, 3 4 5 8 9 10 12 13 14 16 17 18 19 and 6 (31.5%) had either temporary syrinx drainage or shunting 4 10 11 15 16 ; 7 had arachnoid cyst fenestration or removal, 5 7 8 12 13 15 17 and finally, duraplasty was performed in 8 (42.1%) patients. 2 3 14 16 17 19 Five patients received a second intervention. 4 13 15 Neurological and pain improvements were reported in 11 patients during the early postoperative course, 2 3 8 10 11 12 14 15 17 19 23.5% of whom remained unchanged, 5 9 18 and 4 worsened. 4 13 16 Two patients, one from our study, showed neurological improvement after rehabilitation. 13 Wang et al reported worsening after the first improvement during the long-term postoperative course. 15 A total CSF block on CT myelogram 4 17 or Cine MRI 16 was confirmed in three reports ( Table 1 ).

**Table 1:** Cases of arachnoiditis ossificans with syringomyelia reported in the literature

Study, year	Age/Gender	Preceding events (the year before)	Symptoms/Signs	MRI/CT	AO site/type	Surgical treatment	Outcome	F/U (months)
Papavlasopoulos et al, 2006 7	30/M	Perinatal hypoxia, neonatal meningitis	LBP, PP, hyperreflexia, Babinski, hypoesthesia to pain, light touch, and temperature below Th 10	AO: Lower thoracal, lumbar, and cauda equina, intradural extramedullary cyst Th 9–11 dorsally SM: T 10–12	Thoracolumbar/II and III	Laminectomy Th 8–10, cyst drainage	Unchanged	–
Singh et al, 2010 17	81/F	–	LBP, PP, dysesthesia pain, urinary incontinence, Th 8 sensory level, Babinski	Central disc Th 6/7, SM: Th 6 to conus, dorsal intradural arachnoid cyst, complete block below Th 8	Thoracic/–	Laminectomy Th 6–8, subarachnoid cyst resection, AO resection partially, right costotransversectomy, duraplasty	Improved	12
Hasturk et al, 2013 8	48/F	AS (10), dorsal stabilization Th 12-L1, 2, 4, 5, Smith–Peterson osteotomy L3-4(8)	LBP. PP, urinary dysfunction, hypoesthesia below Th6, diminished anal tonus	SM: Th 6–12, AO: Th 9–12, intradural extramedular cyst Th 10–12	Thoracic/I	Laminectomy Th 9–11, cyst drainage, AO dissection	Improved	–
Kahler et al, 1998 11	62/M	SAH (15)	LBP, quadriparesis, hyperreflexia, impotency, Babinski, sensory level below Th 5	SM: C7-Th 9, AO: Th 2–11	Thoracic/I	Laminectomy Th 6–7, syringopleural shunt	Improved	3
Nagpal et al, 1975 4	39/M	Treated for STD	Quadriparesis, spastic paraplegia, a sensory level below C3 left side and Th4 right side, Babinski	SM: Cervical and thoracic with CSF block at Th 4 and T11	Thoracic/–	First OP: Laminectomy Th 4–7, removed AO, Second OP: Laminectomy Th 7–9, silastic tube syringosubdural shunt	Worsened, ended up with syringobulbia	–
Mello et al, 2001 9	49/F	Surgical removal intradural extramedullary meningioma (24) with residual right-sided dominant paraparesis	PP, urinary incontinence, hyperreflexia, hypesthesia below Th 10	Intramedullary cyst 2 cm at Th 10, increased in size 1 year later	Thoracic/–	Laminectomy Th 10 AO removal	Unchanged	12
Opalak and Opalak, 2015 10	62/M	Quadriplegia due to trauma, ACDF C4-6 (34), intrathecal Phenol therapy	Dysautonomia	SM: Th 6-conus, AO: Th 6-conus	Thoracic/I and II	Laminectomy Th 11–L2, AO removal, shunting from conus	Improved	−
Ibrahim et al, 2010 3	36/M	Resected cerebellar ganglioma complicated with postoperative CSF leakage (17)	PP, gait ataxia, positive Romberg's test	SM: Th 3, intradural extramedullary mass Th 4–5, hypointense nodularity Th 5–10 posteriorly	Thoracic/–	Laminectomy Th 4–7, AO dissection, duraplasty	Improved	9
Domenicucci et al, 2004 2	63/F	Meningitis (49)	LBP, PP, urinary retention	SM: Th 2–11, AO: Th 3–11	Thoracic/I	Laminectomy Th 3–10, duraplasty	Improved	6
Slavin et al, 1999 16	54/F	–	Pain left chest side, PP, sensory loss below Th 8, Babinski left	SM: Th 7 to conus, venous malformation, CT postop. Circumferential AO Th 6–9	Thoracic/II, I	Laminectomy Th 9–11, AO removal, venous malformation coagulated, myelotomy, syringoperitoneal shunting, duraplasty	Worsened, then improved	6
Wang et al, 2019 15	70/F	Spontaneous spinal hemorrhage and oil-based contrast myelogram (30)	Pain right side chest allodynia, PP, ataxia	Thoracic SM, tethering of spinal cord at Th 7, thoracolumbar AO Th 5–11, arachnoid cyst	Thoracolumbar/I, II (Th 5–11)	First OP: Laminectomy Th 5–8, fenestration of the arachnoid cyst, adhesiolysis. Second OP: Syringopleural shunt	Initially, improved, then worsened	12
Revilla et al, 1999 13	68/f	SAH (14)	LBP, PP, difficulty with urination	SM: Cervicomedullary to conus, extramedullary arachnoid cyst, AO: Th 5–9	Thoracic/I	First OP: Laminectomy Th 3–T4, cystotomy Second OP: laminectomy Th 2–5, AO removal	Worsened, then minimally improved	24
Wang et al, 2017 18	41/F	Unknown	PP, sensory deficit below Th8, hyperreflexia	SM Th 6–11, AO: Th 5–12	Thoracic/I	Laminectomy Th 6–8, partially AO removal	Unchanged	24
Van Paesschen et al, 1990 5	34/F	Tuberculous meningitis (20)	LBP, gait disturbance, PP, urinary urgency, Babinski, sensory disturbance	No CSF block in CT myelogram, SM Th 5–10, intradural extramodular cyst, AO	Thoracic/I	Laminectomy Th 5–9, cystectomy, AO removal	Unchanged	12
Miloš Joković et al, 2019 14	56/F	combat-related injury (27), trauma with fracture vertebrae Th 4, 5 (24), thyroiditis Hashimoto Multifocal motor neuropathy	Progressive tetraparesis, hyperreflexia, Babinski, decreased sensation below Th 4/5	AO: Th 4–5, SM: cervicothoracic	Thoracic/I	Laminectomy Th 4–5, AO removal, duraplasty	Improved	14
Bailey et al, 2021 12	58/F	–	Right shoulder pain, right-hand numbness, and muscle fatigue in bilateral legs	SM: C4-Th 1, Th 3–10, AO: Th 2-L2, arachnoid cyst	Thoracolumbar/–	Th 2–T9 laminectomy, fenestration arachnoid cyst, AO removal	Improved	–
Nagashima et al, 2022 19	66/M	AH (12)	Gait disturbance and dysuria	SM: Th 5–8, tethered spinal cord Th 8–10, CSF block in myelogram, AO upper thoracic to Th 9	Thoracic/I	Duraplasty and expanding laminoplasty Th 6–9, AO removal, arachnoidolysis	Improved	–
Our patient, 2018, Case 1	72/F	Meningitis (55)	LBP, PP, hyperreflexia	SM: Th 3–10, AO: Th 5–10, CSF block in CT myelogram and Cine MRI at Th 8	Thoracic/I	First OP: Laminectomy Th 7–8 Second OP: laminectomy Th 5–10, duraplasty	Worsened initially, improved after second OP	36
Our patient 2018, Case 2	72/M	Epidurals, spondylodiscitis (16)	LBP, PP, hyperreflexia	SM: Th 4–7, AO: Th 4–12	Thoracic/I&II	First OP: Laminectomy Th 6–7 Second OP: Laminectomy Th 8 and duraplasty	Progressive PP	36

Abbreviations: ACDF, anterior cervical discectomy and fusion; AO, arachnoiditis ossificans; AS, ankylosing spondylitis; F, female; F/U, follow-up; LBP, low back pain; M, male; mon, month; OP, operation; PP, progressive paraparesis; SAH, subarachnoid hemorrhage; SM, syringomyelia; STD, sexually transmitted disease

## Discussion

**Fig. 4:**
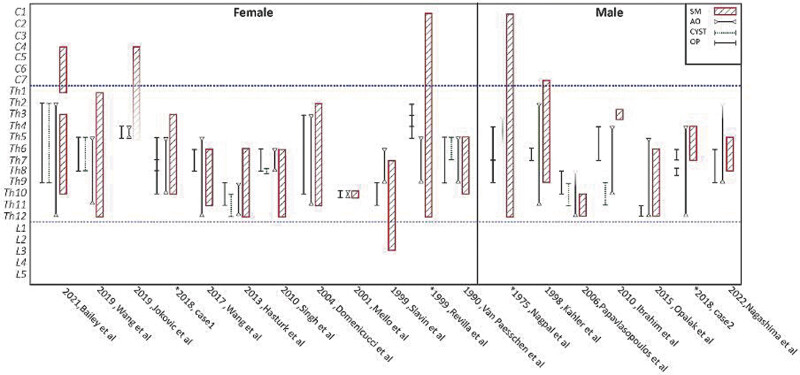
Localization of AO, SM, arachnoid cysts, and the operation sites in both genders. AO, arachnoiditis ossificans; SM, syringomyelia. *Patients who underwent two surgeries.

AO was described before the MRI era through autopsies 20 and intraoperative exploration. 4 According to Burton, AO is thought to be a sequela of chronic AA, the final stage of the three-stage inflammatory process. 21 Another theory for the development of AO is related to a cluster of arachnoid cells commonly located in the lower thoracic area. 22 Poor vascularization leads to age-related degenerative changes, resulting in bony metaplasia. 20 23 Inflammatory cells are histologically absent. 4 24 The coincidence of SM with AO was reported in 17 peer-reviewed case reports. 5 AO-SM is primarily located in the thoracic spine. The exact mechanism by which SM is associated with AO is unclear. The development of SM is believed to be secondary to that of AO. Therefore, scarring due to inflammatory processes in the leptomeninges compromises the spinal cord's local vascular supply, leading to ischemia in the poorly vascularized central region. Finally, tissue necrosis causes cavitation. 11 25 26 The progression of SM is supposedly a result of altered CSF flow dynamics, subsequent dissociation of spino-spinal pressure, and expansion of SM. 11 26 The SM is mainly localized close to the AO level, possibly with rostral or caudal expansion ( Fig. 4 ). SM associated with arachnoiditis was classified as a non-communicating isolated central canal SM. Histologically, in 23 autopsied patients, SM depicted complex cavity areas of ependymal denuding and paracentral dissection into the spinal cord parenchyma. Moreover, most isolated SM end at the upper end due to central canal stenosis. 27 SM has been reported in other similar clinical scenarios, such as AA, 28 arachnoid webs (AWs), 29 arachnoid cysts, 30 and, rarely, arachnoid telangiectasia, 31 which might support the pathological mechanisms of impaired CSF dynamics leading to the development of SM.

The presumed predisposing factors of AO include infection, trauma, intrathecal injections, spinal surgeries, and, rarely, subarachnoid hemorrhage. Many authors agree that a combination of many possible causative events might lead to the osteoblastic proliferation of the meningioendothelial system. 2 23 Many authors have discussed the underlying mechanism of the development of SM, but until recently, the cause has remained unclear. Domenicucci et al described the most accepted morphologic classification in 2004. The patients were presented with myelopathy and mild to severe symptoms typical of SM. Non-enhanced CT provides the best visualization of the AO and is highly sensitive. Sagittal and axial reconstruction should be performed to classify the AO. MRI with and without contrast enhancement is the best method for detecting the syrinx and ruling out the associated pathologies. MRI revealed AO with a hypointense signal dorsal to the spinal cord. Cine MRI and conventional CT myelograms can supply additional information by depicting a CSF block. Ossified tissue of the meninges is mainly described histopathologically as lamellar bone.

Here, we will discuss the differential diagnosis, chronological view, and surgical approaches for SM with AO.

### Differential Diagnosis

An AW can be associated with arachnoid cysts or, rarely, with spinal cord herniation. It is, however, most often idiopathic. Coincidence with a syrinx has seldom been reported. The scalpel sign observed in the sagittal MR T2W image is typical. The diagnosis was confirmed intraoperatively. 29 AW tends to be thoracic and dorsal to the spinal cord. Histologically, fibrous tissue accumulated in layers with no lymphocytes or dyskaryotic cells. AW causes an incomplete obstruction with a one-way valve mechanism, either caudal or rostral. This is why the syrinx is either caudal or rostral to the AW. Surgical interventions for symptomatic patients include fenestration and resection of the web, which provides neurological and functional improvements. 32 In a case series, the syrinx collapsed or diminished in size postoperatively. This finding supports the assumption that disturbances in CSF flow dynamics due to arachnoiditis or mechanical disturbances are causal. 32

However, AA tends to develop after a severe inflammatory intradural process. The coincidence of SM has been described in many studies. In a case series, Zuev et al reviewed 47 patients with AA and SM. 33 The preceding events were intradural spinal surgery, meningitis, peridural anesthesia, spondylitis, and subarachnoid hemorrhage. There were no data on previous inflammation in 19 patients. Thirty-four patients underwent surgery with a total of nine surgeries involving either syringopleural, syringoperitoneal, or syringosubarachoidal shunting. CSF circulation was restored with or without sharp dissection of the adhesions, and duraplasty was subsequently performed to prevent spinal cord tethering. Syrinx progressed by 20.6% postoperatively. Depending on the severity of the adhesion, 50% of severe patients achieved clinical stabilization after 1 year. The size of the syrinx continued to increase in 50% of patients. The incidence of poor long-term outcomes after bypass surgery was 66.7%. 33

#### Surgical Approaches

The surgical management of AO-SM patients is widely heterogeneous ( Table 2 ). Koyanagi et al provided a case series of 15 patients in their clinic between 1982 and 2000 who suffered from arachnoiditis and a syrinx and underwent a variety of surgical procedures. The causes of spinal arachnoiditis were meningitis in nine patients, spinal surgery in two patients, and no complications in four patients. Clinical features at admission included either tetraparesis or paraparesis. Total CSF blockage was confirmed in 11 patients. Surgical management consisted of the placement of a syringoperitoneal shunt (10 patients), a syringosubarachnoid shunt (3 patients), or a ventriculoperitoneal shunt (2 patients) as the initial surgical treatment. Eight patients underwent further shunt operations 2 months to 12 years after the first surgery because of the progression of paraparesis, tetraparesis, or re-expansion of the syrinx. The rate of neurological improvement was reported to be 60%. The case series provided neither native CT nor pathological findings. Intraoperative AA has been described since most patients are affected by either tuberculous meningitis or meningitis. 28

**Table 2:** Different surgical approaches and outcomes of patients

Approach a	Outcome		
	Improved	Worsened	Unchanged
Cystectomy	–	–	1
AO resection ± cystectomy	2	1	3
Duraplasty	3	1	–
AO resection + duraplasty ± cystectomy	4	–	–
SM shunting	1	–	–
AO resection + SM shunting	1	1	–
AO resection + myelotomy + SM shunting + duraplasty		1	–
Sum	11	4	4

Abbreviations: AO, arachnoiditis ossificans; SM, syringomyelia.

a Laminectomy was performed on all patients.

Mallucci et al, on the other hand, provided a case series describing idiopathic SM treated with laminectomy and re-establishment of normal CSF flow with duraplasty with more success. 34 In this series, only two patients who underwent shunting deteriorated clinically. This study supported Williams's theory, which suggested that if such a partial block exists within the spinal subarachnoid space, then pressure gradients may be created across the block, encouraging the movement of CSF into the cord. Once such collections have been established, they may be maintained until the mechanism causing the block is removed. The author recommended that the treatment of choice for idiopathic SM is laminectomy and excision of the obstructive arachnoid lesion, leading to the re-establishment of normal CSF flow and, thus, elimination of the filling mechanism.

In 1995, Sgouros and Williams wrote an article about the long-term outcome of shunting and draining SM. 35 After 10 years of follow-up, only 53.5% of patients with syringopleural and 50% of patients with syringosubarachnoid shunting remained clinically stable. A 15.7% complication rate, such as shunt blockage or dislodgement, low-pressure symptoms, and CSF fistula for syringopleural shunting, was recorded. Syringosubarachnoidal shunts have been shown to cause subarachnoid hemorrhage, blockage, implantation failure, infection, and overdrainage, which are common complications. The authors concluded that treating and re-establishing the filling mechanism of the syrinx is more critical than draining the syrinx cavity itself. Myelotomy (syringotomy) can cause immediate neurological damage. The data on the success of ventriculostomy are limited. In this series, patients with non-traumatic spinal arachnoiditis deteriorated after syringopleural shunting clinically in the short and long term during the postoperative course. Additionally, shunting of the syrinx was performed in five patients, three of whom had poor outcomes. 4 15 16

A glance at the surgical approaches of patients with AO-SM showed that cystectomy with or without AO dissection did not provide a clear benefit. According to the reported studies, clinical improvement was observed in four patients, 8 10 14 17 three patients experienced no clinical change, 5 9 18 and clinical deterioration was reported in three patients. 4 15 16 On the other hand, duraplasty alone or as an additive for AO removal was superior to different approaches, including SM shunting ( Table 2 ). The spinal cord under AO is fragile. The act of manipulation to remove the AO, even in microsurgical techniques, could injure the spinal cord more. Our patient developed a new sensory-motor impairment after undergoing a biopsy. The neurological improvement and stabilization in the long-term postoperative course and the unaltered SM size might confirm the superiority of impaired cerebrospinal fluid dynamics as a leading role in the pathogenesis of AO in the development and progression of SM.

## Conclusion

AO-SM may be misdiagnosed because of the chronic natural course of the disease and because of its similarity to other chronic pain syndromes. We propose that in all arachnoidopathies with CSF circulation blockage that present with myelopathy, SM, and clinically progressive neurological impairment, surgery should be considered. The decision about the appropriate time for a surgical approach, as well as which surgical approach is best, should be made individually. The optimal treatment is not known. A complete set of radiological investigations should be performed to make a surgical decision. Apart from native CT and MRI, we suggest performing either a CT-myelogram or Cine MRI to determine the extent of CSF blockage. Nevertheless, some authors believe myelography could be misleading or non-diagnostic. 36

Although the prognosis following surgical intervention may be unfavorable, such procedures can still serve to stabilize neurological function in certain cases. AO resection entails a significant risk of injury to neural tissue, and the outcome may often be suboptimal. Recent studies indicate that duraplasty could potentially yield a higher success rate, as many patients either exhibit improvement or remain stable after surgery. The efficacy of shunting remains uncertain and may be more appropriate for recurrent cases, given the high rates of deterioration associated with it. Ongoing research and long-term follow-up are essential for the ongoing refinement of treatment strategies for AO-SM.
